# Nanoscale Quantitative Imaging of Single Nuclear Pore Complexes by Scanning Electrochemical Microscopy

**DOI:** 10.1021/acs.analchem.4c01890

**Published:** 2024-06-21

**Authors:** Ran Chen, Pavithra Pathirathna, Ryan J. Balla, Jiyeon Kim, Shigeru Amemiya

**Affiliations:** † Department of Chemistry, University of Pittsburgh, Pittsburgh, Pennsylvania 15260, United States; ‡ School of Chemistry and Chemical Engineering, Southeast University, Nanjing 211189, China; § Department of Chemistry and Chemical Engineering, Florida Institute of Technology, Melbourne, Florida 32901, United States; ∥ Department of Chemistry, The University of Rhode Island, Kingston, Rhode Island 02881, United States

## Abstract

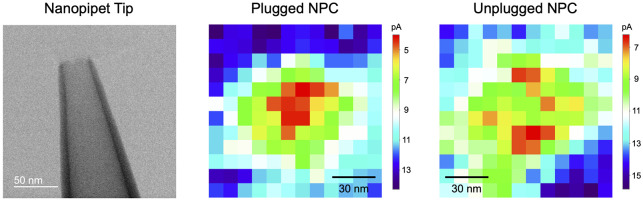

The nuclear pore complex (NPC) is a proteinaceous nanopore that solely and selectively regulates the molecular transport between the cytoplasm and nucleus of a eukaryotic cell. The ∼50 nm-diameter pore of the NPC perforates the double-membrane nuclear envelope to mediate both passive and facilitated molecular transport, thereby playing paramount biological and biomedical roles. Herein, we visualize single NPCs by scanning electrochemical microscopy (SECM). The high spatial resolution is accomplished by employing ∼25 nm-diameter ion-selective nanopipets to monitor the passive transport of tetrabutylammonium at individual NPCs. SECM images are quantitatively analyzed by employing the finite element method to confirm that this work represents the highest-resolution nanoscale SECM imaging of biological samples. Significantly, we apply the powerful imaging technique to address the long-debated origin of the central plug of the NPC. Nanoscale SECM imaging demonstrates that unplugged NPCs are more permeable to the small probe ion than are plugged NPCs. This result supports the hypothesis that the central plug is not an intrinsic transporter, but is an impermeable macromolecule, e.g., a ribonucleoprotein, trapped in the nanopore. Moreover, this result also supports the transport mechanism where the NPC is divided into the central pathway for RNA export and the peripheral pathway for protein import to efficiently mediate the bidirectional traffic.

A greater understanding of molecular transport through the nuclear pore complex (NPC) is fundamentally significant in biology and urgently required in biomedicine.^1^ The NPC is a natural proteinaceous nanopore that solely transports both small molecules and macromolecules between the nucleus and cytoplasm of a eukaryotic cell. The NPC is crucial to the regulation of gene expression^2^ and is linked to many human diseases, including cancers,^3^ neuronal diseases,^4^ and viral infections.^5^ Moreover, the NPC is an attractive target to mediate the nuclear import of various therapeutic macromolecules and nanomaterials for gene therapy of many human diseases and genetic disorders.^6^ This chemical task, however, is highly challenging because the transport barriers of the NPC prevent the passive transport of large substances (>40 kDa).^7^ Biologically, importins can proficiently chaperon ∼1000 copies of passively impermeable nuclear proteins through each NPC every second,^8^ while RNA export is facilitated by exportins.^9^ It, however, is not well understood how the nanopore mediates the efficient bidirectional traffic, thereby requiring new experimental approaches to reveal the mechanism.^10^

Herein, we image single NPCs by nanoscale scanning electrochemical microscopy^11,12^ (SECM) to address a long-standing question about the central plug located in the ∼50 nm-diameter pore.^13^ Specifically, we demonstrate that nanoscale SECM uniquely determines the lowered permeability of the plugged NPC compared to the unplugged NPC to a small probe ion (Figure 1A). Nanoscale SECM imaging supports that the central plug is not an intrinsic transporter^13^ but is a macromolecule, specifically, ribonucleoprotein (RNP), captured in the pore to block molecular transport.^14^ These two origins have been debated for decades^13−15^ and cannot be distinguished directly by the structural imaging of the central plug with cryo-electron tomography^16^ and atomic force microscopy (AFM).^17^ Upon treatment with RNase, plugs changed in shape and decreased in volume as imaged by AFM,^18^ thereby supporting the RNP-based plug although the volume decreased only by 18%. The RNP-based plug is consistent with the transport mechanism^19^ where the NPC pore is concentrically divided into central and peripheral pathways to efficiently mediate RNA export and protein import, respectively.^20−23^

**Figure 1:**
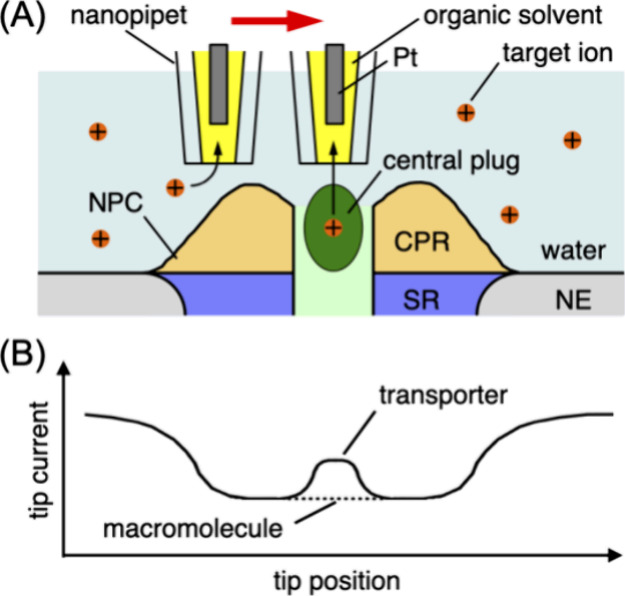
(A) Constant-height SECM imaging with a nanopipet tip scanned over the cytoplasmic ring (CPR) of the NPC in the nuclear envelope (NE). SR represents the spoke ring of the NPC. (B) Tip current when the central plug is a permeable transporter (solid line) or an impermeable macromolecule (dotted line).

Experimentally, we resolve the passive permeability of plugged and unplugged NPCs to a probe ion, i.e., tetrabutylammonium (TBA^+^), by employing the SECM tips based on nanopipet-supported interfaces between two immiscible electrolyte solutions^24^ (Figure 1A). With this nanotip, a Pt electrode in the internal organic electrolyte exerts a bias across the nanotip-supported liquid–liquid interface against an electrode in the aqueous solution (not shown) to yield the amperometric tip current based on TBA^+^ transfer. The current response is lowered as the tip moves laterally toward the cytoplasmic ring^25^ (CPR) (Figure 1B), which hinders the diffusion of the probe ion to the nanopipet tip. We demonstrate that the tip current is suppressed or recovered over the pore with or without a plug (solid and dotted lines, respectively), which is impermeable to the probe ion. We also find that an extremely short tip–NPC distance is required to recover the tip current over the unplugged pore of the nuclear envelope (NE) supported on the impermeable glass plate.

The outcomes of this work are biologically relevant, although the NE is isolated, supported on a glass plate, and chemically fixed to facilitate nanoscale SECM imaging. The NPCs of the solid-supported NE mediate importin-facilitated macromolecular transport, as expected physiologically,^35^ and maintain the dynamics of transport barriers, despite chemical fixation.^17^ We ensure in this work that the chemical fixation does not alter the permeability of the NPCs to TBA^+^. Moreover, our previous SECM studies demonstrated that the passive permeability of the NPCs to small probe molecules is determined by the pore size and is not affected by the redox activity, charge, and hydrophobicity of the probe molecules.^19^ These previous studies also demonstrated that the identical permeability of the NPCs to a charged ferrocene derivative was obtained by using metallic tips and organic-filled pipet tips, thereby excluding the effect of the organic solvent leaching from the pipet on the pore permeability.^19^

More broadly, this work employs 25 nm-diameter nanopipet tips to represent the highest-resolution nanoscale SECM imaging of biological samples. Nanoscale liquid/liquid interfaces can be formed reproducibly and reliably at the tip of a well-characterized nanopipet, in contrast to solid nanotips,^26,27^ which can be damaged by electrostatic charge.^28^ Nanopipet tips with ∼30 nm diameters were employed originally to image molecular transport through single solid-state nanopores.^29,30^ Recently, nanopipet tips enabled the detection of acetylcholine released from single neuronal clafts.^31,32^ These studies, however, did not report any SECM images and relied on optical microscopy to position the nanotip at the neuronal claft. More recently, ion-selective nanopipets were employed to image the release of lactate from single living bacterial cells.^33,34^ These studies, however, employed larger ∼120 nm-diameter nanopipets.

## Experimental Section

### Chemicals and Materials

Tetradodecylammonium (TDDA) bromide, 1,2-dichloroethane (DCE; >98%), tetrabutylammonium chloride, and *N*,*N*-dimethyltrimethylsilylamine (Selectophore grade) were purchased from Sigma-Aldrich (St. Louis, MO). Potassium tetrakis(pentafluorophenyl)borate (TFAB) was obtained from Boulder Scientific (Mead, CO). All of the reagents were used as received. The TFAB salt of TDDA was prepared by metathesis.^36^ Buffer solutions were prepared with 18.3 MΩ·cm deionized water obtained from a Milli-Q Advantage A10 water purification system (EMD Millipore, Billerica, MA). A TOC value of 2–3 ppb was measured by using an internal online TOC monitor. The Milli-Q system was fed with water (15.0 MΩ·cm) purified from tap water by using Elix 3 Advantage (EMD Millipore). The resulting solutions were passed through a 0.02 μm filter (Whatman Anotop syringe filter, Sigma-Aldrich) before use for nanoscale SECM imaging.

### Nanoscale SECM Instrumentation

**Figure 2:**
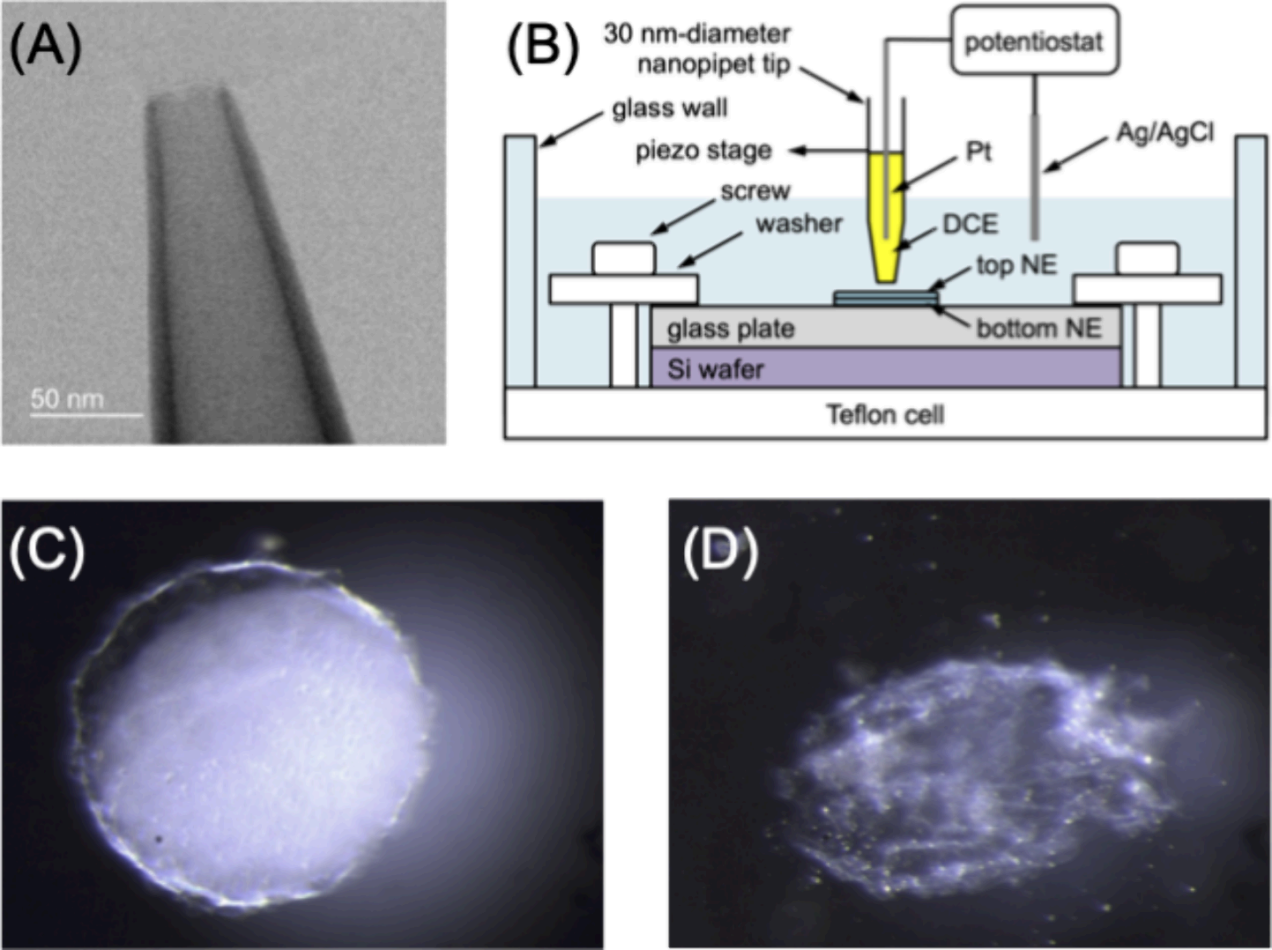
(A) TEM image of a quartz nanopipet. (B) SECM setup with a nanopipet tip over the cytoplasmic side of glass-supported double NEs. Photos of (C) the isolated nucleus and (D) the NE spread on the glass support.

A home-built SECM instrument^29^ with an isothermal chamber^37^ was used for nanoscale imaging. Electrochemical measurements were carried out using a commercial potentiostat (CHI 900A, CH Instruments, Austin, TX). Quartz nanopipet tips were fabricated as detailed elsewhere.^30^ Briefly, a quartz capillary (O.D. 1 mm, I.D. 0.7 mm, 10 cm long, Sutter Instrument, Novato, CA) was air-blow cleaned before pulling and was pulled in a CO_2_-laser puller (Model P-2000, Sutter Instrument). Approximately 25 nm-diameter nanopipets were obtained reproducibly by running the line of a standard program with parameters of heat = 700, filament = 4, velocity = 60, delay = 145, and pull = 125. The pipet diameter was confirmed by transmission electron microscopy (JEM-2100F, JEOL USA, Peabody, MA; Figure 2A), as detailed in the Supporting Information. A nanopipet was reacted with *N*,*N*-dimethyltrimethylsilylamine, filled with a DCE solution of 0.1 M TDDA–TFAB, and immersed in a buffer solution for nanoscale SECM imaging (Figure 2B). The buffer solution contains 5 mM TBACl and 0.55% PVP. Pt and Ag/AgCl wires were placed inside and outside of an organic-filled nanopipet, respectively.

### Preparation of Supported NE

The glass-supported double NE (Figure 2B) was prepared as described below for AFM and nanoscale SECM imaging. We employed a glass slide to support the nucleoplasm-free double NE of a large nucleus (∼0.4 mm in diameter) isolated from the stage VI oocyte of a *Xenopus laevis* frog.^38^ Oocytes were extracted from the ovary cluster of an adult female frog^39^ (NASCO, Fort Atkinson, WI) and stored at 18 °C for less than 3 days before use. The nucleus was isolated from the oocyte in the isotonic 1.5% poly(vinylpyrrolidone) (PVP) solution of mock intracellular buffer (MIB) at pH 7.4. MIB contains 90 mM KCl, 10 mM NaCl, 2 mM MgCl_2_, 1.1 mM EGTA, 0.15 mM CaCl_2_, and 10 mM HEPES.^40^ EGTA was used to mimic a physiological concentration of free Ca^2+^ (∼200 nM) in oocytes.

Specifically, the isolated nucleus was transferred on a glass slide treated with Cell Tak (BD Biosciences, Bedford, MA) as a biological adhesive (Figure 2C). The nucleus was swollen in a hypotonic MIB solution containing 0.55% PVP to detach the NE from the nucleoplasm. The nucleoplasm was removed by using minute pins under a stereomicroscope (SZX-ZB7, Olympus, Center Valley, PA). Subsequently, the top part of the NE collapsed on the bottom part,^41^ thereby yielding the double NE on the glass slide (Figure 2B,D). The hypotonic MIB solution was replaced with PVP-free MIB as low Ca^2+^ media to maintain plugs or with nuclear isolation media (NIM) as high Ca^2+^ media to remove plugs.^42^ NIM contained 1.5 mM CaCl_2_, 87 mM NaCl, 3 mM KCl, 1 mM MgCl_2_, and 10 mM HEPES at pH 7.4. The high or low Ca^2+^ media was exchanged with the same media containing 2.5% glutaraldehyde^17^ to fix the NEs. The fixed samples were washed with water and dried overnight in ambient air.

## Model

**Figure 3:**
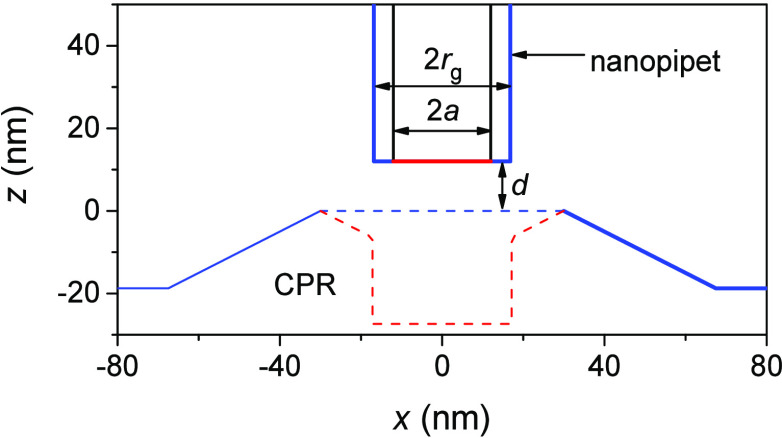
*x*,*z* cross-section of the model at *y* = 0 for an SECM diffusion problem at a nanopipet-supported tip over an unplugged and plugged NPC. The red dashed line represents the inner wall of the unplugged pore. The blue dashed line represents the impermeable plug that blocks the pore.

The SECM images of single NPCs were compared to those simulated by employing the finite element method based on COMSOL Multiphysics (version 6.2, COMSOL, Inc., Burlington, MA). The current response of a nanopipet tip was calculated by solving a three-dimensional diffusion problem with the cytoplasmic side of an impermeable or permeable NPC in Cartesian coordinates (Figure 3). See the “Complete Report” generated by COMSOL in the Supporting Information for details of simulation geometry and conditions. AFM images (Figure S2) were used to define the exterior geometry of the plugged and unplugged CPRs. Cryo-electron tomographic images of the NPC^25^ were used to define the interior of the unplugged CPR.

The steady-state diffusion of a target ion in the solution and pore was defined by
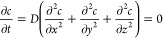
1where *t* is time, *c* is the concentration of the transported ion at (*x*, *y*, *z*), and *D* is the diffusion coefficient of the ion. Initially, the aqueous solution contained a target ion at a bulk concentration of *c*_0_. The zero ion concentration at the pipet tip (red solid line in Figure 3) is used as the surface boundary condition of the directional TBA^+^ transfer from the aqueous solution into the pipet. The ion diffusion in the inner organic solution does not affect the tip current, which is limited by the diffusion of the target ion in the outer aqueous solution. The pore wall and pipet wall are impermeable to the target ion, which correspond to zero perpendicular flux (blue solid lines). A central plug is impermeable and is represented by a blue dashed line. By contrast, the interior space of the unplugged NPC is defined by the impermeable wall (red dashed line). The simulation result was independent of the pore depth, thereby considering a shallower pore than the ∼80 nm-long actual pore.^25^ Simulation space limits are set far away to yield the bulk concentration of the target ion, *c*_0_.

## Results and Discussion

### AFM Imaging of Plugged and Unplugged NPCs

**Figure 4:**
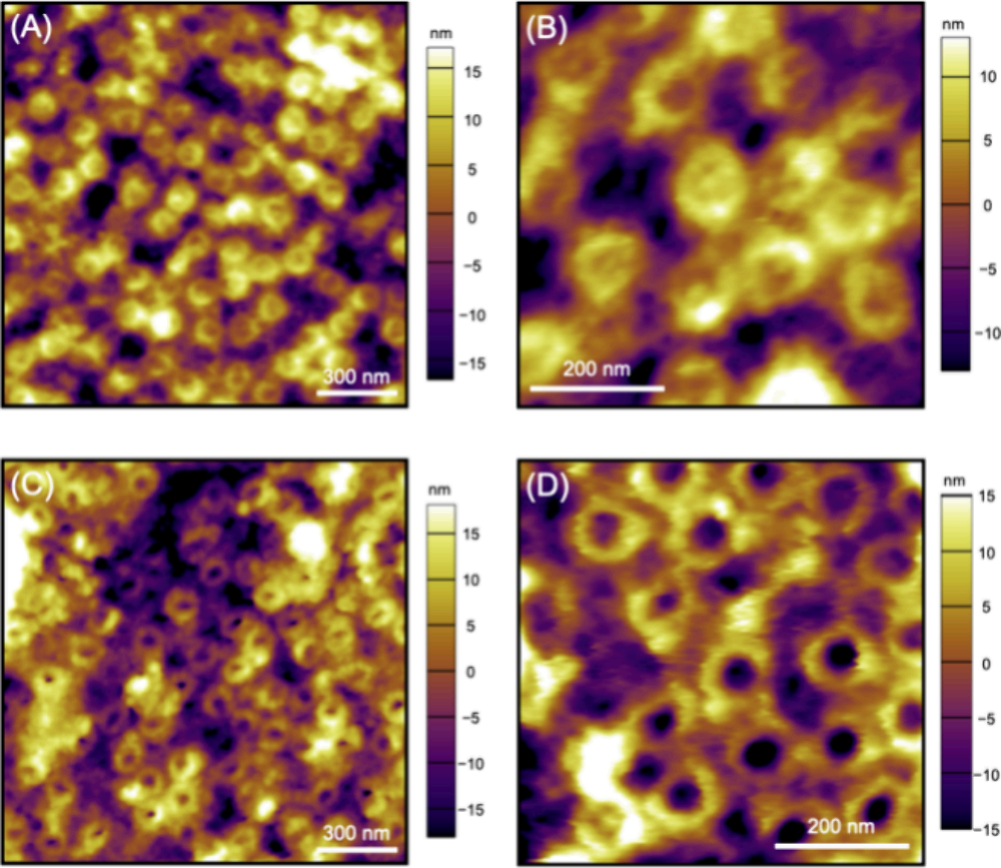
The cytoplasmic side of (A, B) plugged and (C, D) unplugged NPCs, as treated in MIB and NIM, respectively, and imaged in the air by AFM.

We employed AFM to image plugged and unplugged NPCs of the NE (Figure 4), which was isolated from the large nucleus of a *Xenopus laevis* oocyte (Figure 2C,D). The presence and absence of a plug was confirmed by imaging the cytoplasmic side of NPCs. The top part of the NE of the nucleus was stacked on the bottom part (Figure 2B) to image the cytoplasmic side of the NPC by AFM. A plug can not be seen from the nucleoplasmic side of the NPC covered with the nuclear basket.^17^ The NE was fixed with glutaraldehyde to maintain the plugged and unplugged states of the NPCs^17^ without altering their permeability to TBA^+^, as confirmed by microscale SECM (Figure S3). AFM images confirmed that the NPC was plugged or unplugged when the NE was incubated in buffer solutions containing physiological submicromolar or low millimolar concentrations of Ca^2+^, respectively.^22^

Specifically, ∼75% of NPCs were plugged (Figure 4A,B) or tangled^17^ when the NE was incubated in MIB containing a physiological concentration of ∼0.2 μM Ca^2+^ in the oocyte. By contrast, ∼80% of NPCs were neither plugged nor tangled (Figure 4C,D) when the NE was incubated in NIM containing a high concentration of 1.5 mM Ca^2+^. The densities of NPCs treated with MIB and NIM were ∼40 NPCs/μm^2^, which is close to the density determined for the *Xenopus* oocyte nucleus by cryo-electron tomography.^25^ Moreover, the high concentration of Ca^2+^ in NIM removed the central plug without affecting the size of the CPR. AFM imaging, however, can not decide whether the plug is the permeable transporter of the NPC^13^ or an impermeable in-transit macromolecule.^14^

### Nanoscale SECM Imaging of Single NPCs

**Figure 5:**
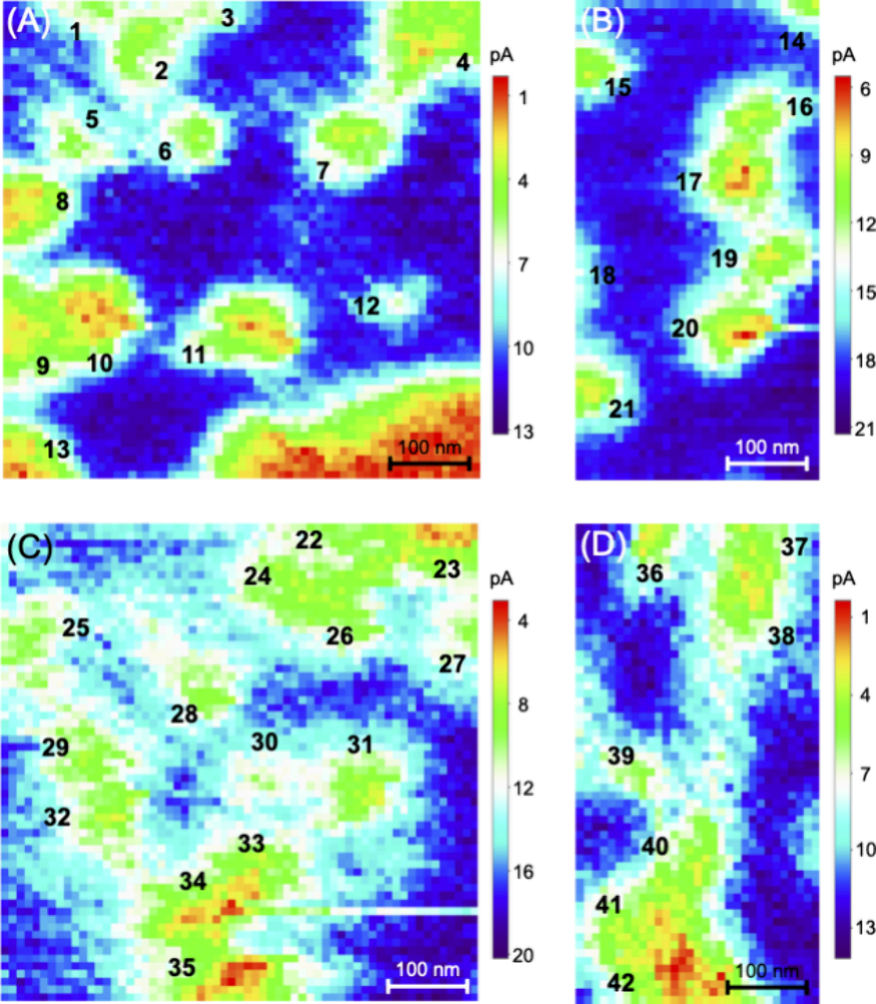
Constant-height SECM images of single NPCs treated in (A, B) MIB and (C, D) NIM containing 5 mM TBACl.

We employed the constant-height mode of nanoscale SECM to image single NPCs successfully (Figure 5). The cytoplasmic side of single NPCs was imaged by scanning an ∼25 nm-diameter pipet tip (Figure 2A) over the NE supported by a glass plate (Figure 2B). The quartz nanopipet was filled with an electrolyte solution of 1,2-DCE and immersed in MIB or NIM to detect TBA^+^ as a probe ion. The tip diameter was characterized in situ by cyclic voltammetry to obtain a diffusion-limited current response in the bulk solution, *i*_T,∞_, as given by
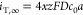
2where *x* is a function of *RG*(43) (=*r*_g_/*a*; *a* and *r*_g_ are the inner and outer radii of a nanopipet tip in Figure 3), *z* is the charge of the detected ion, *F* is the Faraday constant, and *c*_0_ is the bulk concentration of the permeant. The nanopipet tip approached the NE until the tip current decreased to 80% of *i*_T,∞_ before imaging. This current is equivalent to the tip–substrate distance, *d*, of 15.7 nm with the tip radius, *a*, of 12.5 nm.^44^ The tip was scanned laterally at the fixed height, while the tip current was monitored to obtain an SECM image (Figure 5). Each of the numbered NPCs was identified by checking line scans from SECM images as discussed below.

Constant-height SECM images of MIB-treated NEs reproducibly resolved individual NPCs (Figure 5A,B), which are mainly plugged or tangled in the AFM images (Figure 4A,B). For instance, 13 NPCs were identified in a 0.6 μm × 0.6 μm image (Figure 5A) to yield a density of 36 NPCs/μm^2^, as determined by AFM. The actual density of the NPC in the imaged region should be slightly higher because some NPCs were not resolved owing to their contact with the nanopipet tip. The tip–NPC contact is indicated by very low tip currents of ∼1 pA at the bottom right corner of the image (Figure 5A). The tip–NPC contact manifested the limitation of constant-height imaging when the substrate is as rough as or rougher than the tip size.^45^ The AFM images (Figure 4A,B) show that the surface topography of the NE varies by ∼30 nm, which is comparable to the tip diameter. Noncontact SECM imaging of single NPCs was facilitated when the imaged area was smaller, e.g., 0.3 μm × 0.6 μm (Figure 5B), where the tip was scanned further from NPCs. The tip–NPC distances were still short enough to image eight NPCs, which correspond to a density of 44 NPCs/μm^2^.

Constant-height SECM images were also obtained reproducibly with NIM-treated NEs (Figure 5C,D), where most NPCs are unplugged, as imaged by AFM (Figure 4C,D). Fourteen NPCs were identified in a 0.6 μm × 0.6 μm image (Figure 5C) to yield a density of 39 NPCs/μm^2^, as expected from AFM images. A 0.3 μm × 0.6 μm image (Figure 5D) identified seven NPCs (39 NPCs/μm^2^), although the nanopipet tip was scanned closer to NPCs to crash into pore **42**.

### Quantitative Analysis of NPC Images

**Figure 6:**
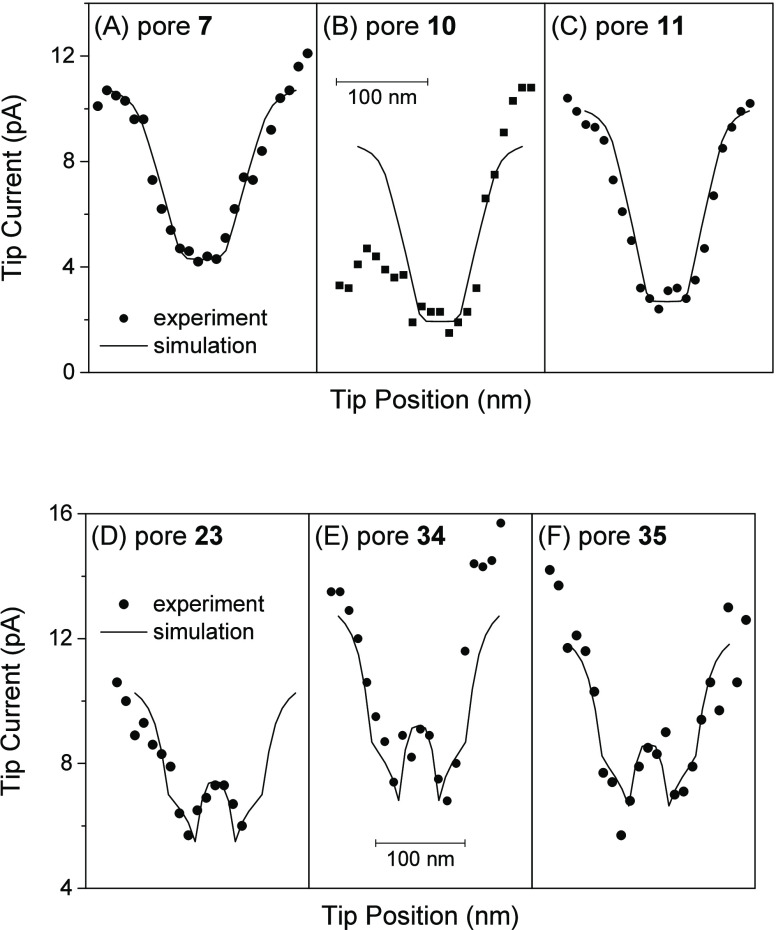
Experimental (dots) and simulated (solid lines) line scans of plugged NPC (A) **7**, (B) **13**, and (C) **14** and unplugged NPC (D) **14**, (E) **25**, and (F) **26** from images presented in Figure 5.

We employed the finite element method to quantitatively demonstrate that individual NPCs were resolved using nanoscale SECM imaging. The line scans of plugged NPCs in MIB fitted well with simulated ones when the plug was assumed to be impermeable to TBA^+^ (Figure 6A–C). The tip current decreased as the tip moved over the center of the NPC. In contrast, the line scans of some unplugged NPCs in NIM were consistent with those simulated by assuming a freely permeable open pore to increase the tip current (Figure 6D–F). Most unplugged NPCs, however, appeared plugged and agreed with the simulations of impermeable pores. This ambiguity is also seen in SECM images (Figure 5C,D) and attributed to variations in the tip–NPC distances (see below). Specifically, we fitted an experimental line scan with a simulated one based on the size and geometry of the NPC determined by AFM and TEM (Figure 3). The former imaged the outer surface of the NPC while the latter imaged the interior pore as detailed in the section Model. We also employed the diameters of the nanopipets that were estimated by TEM and confirmed in situ by cyclic voltammetry.

**Figure 7:**
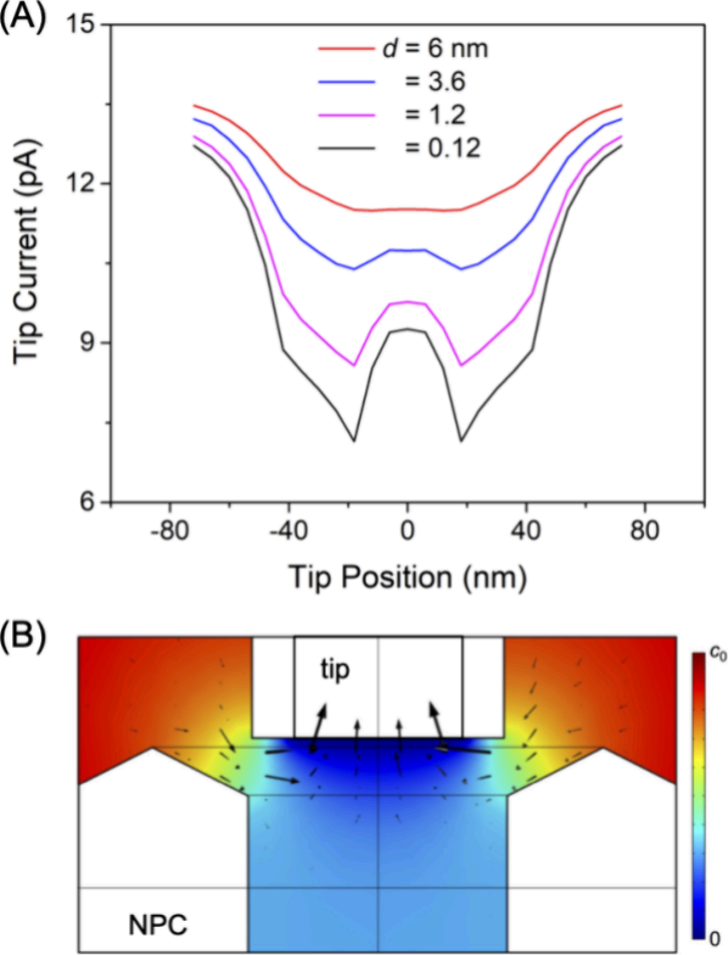
(A) Simulated line scans over an unplugged NPC with a 24 nm-diameter tip at various tip–NPC distances. (B) The concentration profile of the probe ion at *d*/*a* = 0.1. Arrows indicate the flux of the probe ion. See Figure 3 for the dimensions of the tip and the NPC.

We employed the finite element simulation to find also that short tip–NPC distances are required to resolve plugged and unplugged NPCs. The simulated current response of a 24 nm-diameter nanopipet tip increased significantly only when the tip was scanned at 1.2 nm (*d*/*a* = 0.1) or less over the unplugged NPC (Figure 7A). The corresponding concentration profile of the probe ion was also calculated (Figure 7B). The tip current is sensitive to the permeability of the cytoplasmic side of the NPC, where the flux (arrows) is localized. The interior of the pore is uniformly depleted of the probe ion because the nucleus side of the NPC is blocked by the bottom NE and the glass plate (Figures 2B and 3). Subsequently, the minimal flux of the probe ion is provided from the pore interior to the tip.

The control of the short tip–NPC distance for resolution between plugged and unplugged pores is limited by the dynamic oscillation of the piezo stage (Figure 1), which corresponded to a standard deviation of ±0.9 nm.^37^ The resultant fluctuation of the tip–NPC distance significantly affects the tip current (Figure 7A), which results in a deviation between experimental and simulated line scans (Figure 6) to limit the analysis and interpretation of our images.

### SECM Images of Plugged and Unplugged NPCs

**Figure 8:**
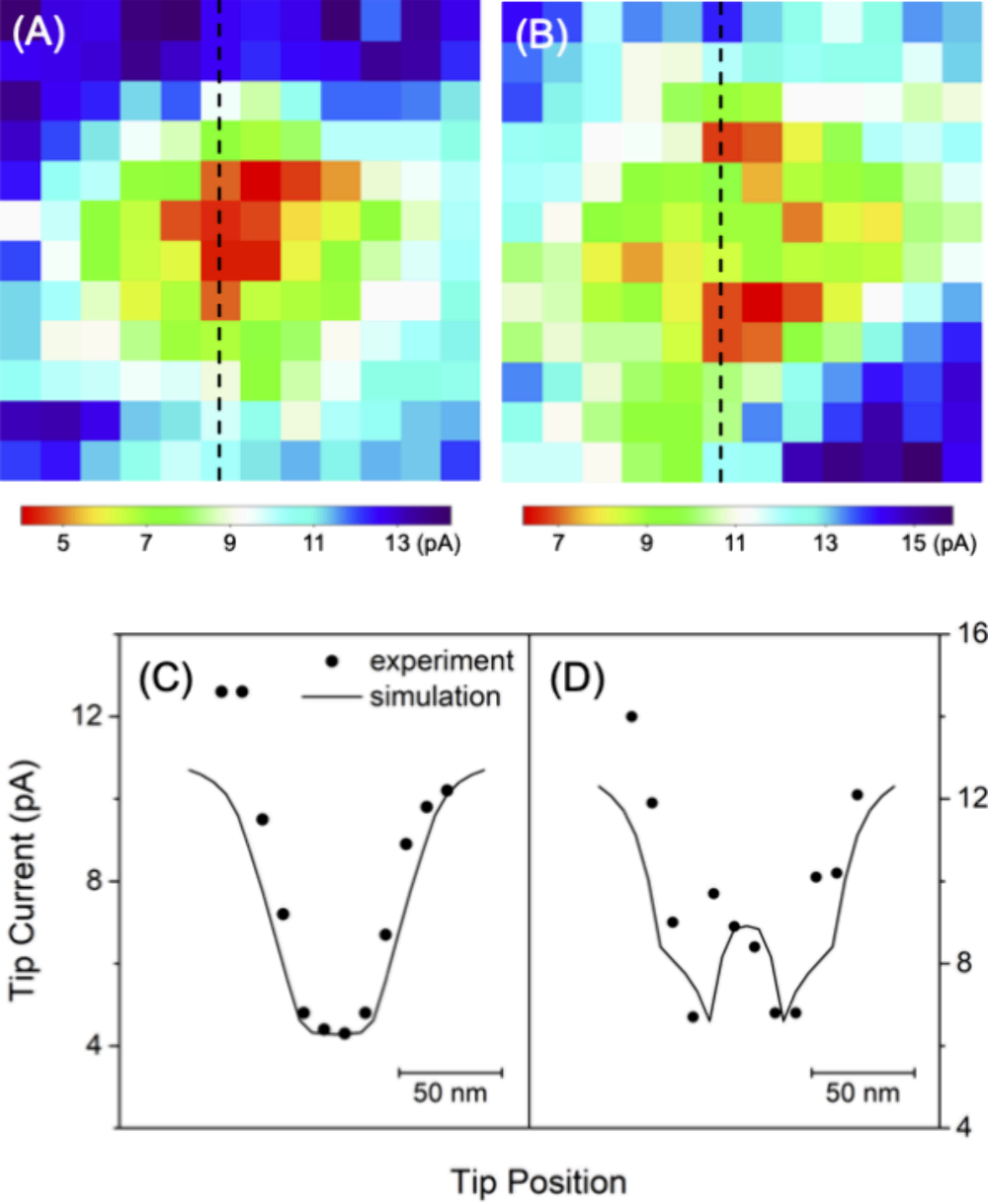
SECM images (120 × 120 nm) of single (A) plugged and (B) unplugged NPCs in MIB and NIM, respectively, containing 5 mM TBACl. Dashed lines indicate the tip positions of line scans for (C) plugged and (D) unplugged NPCs (dots), as fitted with the simulation of impermeable and permeable NPCs, respectively, with *a* = 12 nm and *d* = 1.2 nm (solid lines).

We were able to distinguish between plugged and unplugged NPCs more clearly by constant-height imaging of smaller areas (Figure 8). We were able to maintain short tip–NPC distances of ∼1 nm, as required by the numerical simulation (Figure 7A). The tip current stayed low over a plugged NPC in MIB (Figure 8A), but increased detectably over an unplugged NPC in NIM (Figure 8B). The line scan from the image of the plugged NPC agreed well with the result of the numerical simulation when the pore was assumed to be impermeable to TBA^+^ (Figure 8C). By contrast, the pore of the unplugged NPC was assumed to be freely permeable to TBA^+^, which resulted in a good fit between the experimental and simulated line scans (Figure 8D). These results support the hypothesis that the central plug of the NPC is the impermeable macromolecule^14^ and is not the permeable transporter that is intrinsic to the NPC.^13^ The size of the plug is equivalent to that of RNP^14^ and is large enough to detectably prevent the flux of TBA^+^ driven through the pore by the nanopipet. This result, however, does not mean that the trapped macromolecule completely blocks the flux of TBA^+^ through the pore.^20^ The lowered flux of TBA^+^ is accumulated from ∼30 NPCs under a ∼1 μm-diameter pipet to yield the detectable current response of the larger and more distant micropipet^23^ (Figure S3). The result of the microscale experiment indicates that TBA^+^ can still be transported around the macromolecule trapped in the central pathway, including the peripheral pathway.^20−23^

## Conclusions

In this work, we demonstrated the high significance and power of nanoscale SECM^11,12^ by imaging single biological nanopores. Moreover, we applied nanoscale SECM imaging to address the decades-long question that can not be answered only by structural imaging. Specifically, nanoscale SECM imaging supports the hypothesis that the central plug of the NPC is not an intrinsic transporter but is a blocking macromolecule captured during translocation through the pore. The macromolecule is as large as RNP^14^ and trapped at the center of the NPC pore to constitute a central plug. This result supports our hypothesis that the NPC is concentrically divided into the central pathway for RNA export^21^ and the peripheral pathway for protein import.^20^ The existence of two pathways with complementary roles is significant fundamentally as the mechanism of efficient bidirectional molecular transport through the nanostructured pore. This insight is also relevant biomedically to facilitate ongoing chemical efforts toward the efficient and safe nuclear delivery of genetic therapeutics through the NPCs.^6^

## Supporting Information Available

TEM of nanopipet, AFM analysis of plugged and unplugged NPCs, and permeability of fixed and unfixed NEs (PDF)
Complete Report of finite element simulation by COMSOL (PDF)
