# Supplementary material for: Nanoscale Quantitative Imaging of Single Nuclear Pore Complexes by Scanning Electrochemical Microscopy
Source: Anal Chem. 2024 Jun 21;96(26):10765–71. doi: 10.1021/acs.analchem.4c01890 (PMC11223102; doi:10.1021/acs.analchem.4c01890)
Supplement: Supplementary file 1 — ac4c01890_si_001.pdf [file ac4c01890_si_001.pdf]

Supporting Information

Nanoscale Quantitative Imaging of Single Nuclear Pore Complexes by Scanning Electrochemical Microscopy

Ran Chen,[†,#,‡] Pavithra Pathirathna,[†,f,‡] Ryan J. Balla,[†] Jiyeon Kim,[§] and Shigeru Amemiya[†,*]

[†] Department of Chemistry, University of Pittsburgh, Pittsburgh, PA 15260

[#] School of Chemistry and Chemical Engineering, Southeast University, Nanjing, China

[f] Department of Chemistry and Chemical Engineering, Florida Institute of Technology, Melbourne, FL 32901

[§] Department of Chemistry, The University of Rhode Island, Kingston, RI 02881

[‡] These authors contributed to this work equally.

[*] Email: amemiya@pitt.edu

Table of Contents

TEM of Nanopipet	S-2
AFM Imaging and Analysis	S-4
Permeability of Fixed and Unfixed NEs	S-3
References	S-5

TEM of Nanopipet. A nanopipet was imaged as reported elsewhere.[S-1] A glue was removed from a small piece of carbon tape (16084-1, Ted Pella, Redding, CA) and mounted on the TEM grid (SynapTek DOT, 4510, Ted Pella) by tweezers. A nanopipet was attached to the glue so that the sharp end of the nanopipet was above the slot of the TEM grid (Figure S-1). The nanopipet was cut with tweezers to leave minimum parts remaining on the TEM grid. Objective lens 3 and condenser lens 0 were used for imaging. Objective lens 3 and condenser lens 0 were used for imaging to prevent the deformation of a nanopipet tip.

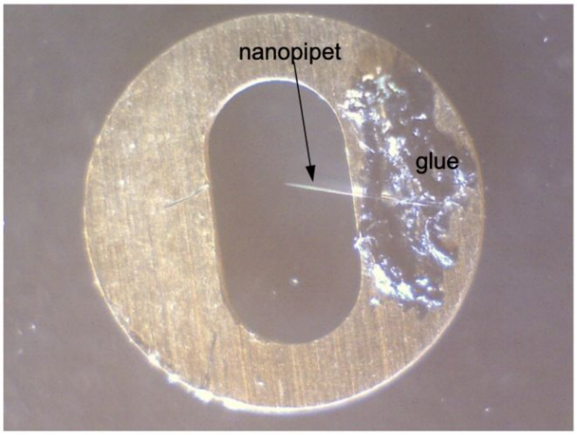

Figure S-1. A 25 nm-diameter nanopipet mounted on a 3 mm-diameter TEM grid.

AFM Imaging and Analysis. The AFM cross-section of each NPC was analyzed to ensure that the high concentration of Ca^{2+} in NIM removed the central plug without affecting the diameter of the CPR, which was close to the diameter determined by cryo-electron tomography.[S-4] Specifically, the highest points of the CPR were identified in AFM images (red dots in Figure S-2) to determine the diameter of the CPR, w, as a distance between the highest points (i.e., the length of the red dashed line). In addition, the highest or lowest point of the plugged or unplugged pore (blue dots), respectively, was determined to define the pore depth, l, as a distance between a dashed line and a blue dot in Figure S-2. Twenty-four plugged NPCs were analyzed to obtain $w = 70 \pm 6$ nm and $l = 2 \pm 1$ nm. By contrast, 20 unplugged NPCs yielded a similar entrance diameter of $r = 70 \pm 10$ nm in addition to a much larger pore depth of $l = 18 \pm 6$ nm, which was limited by the penetration depth of the AFM cantilever.[S-5]

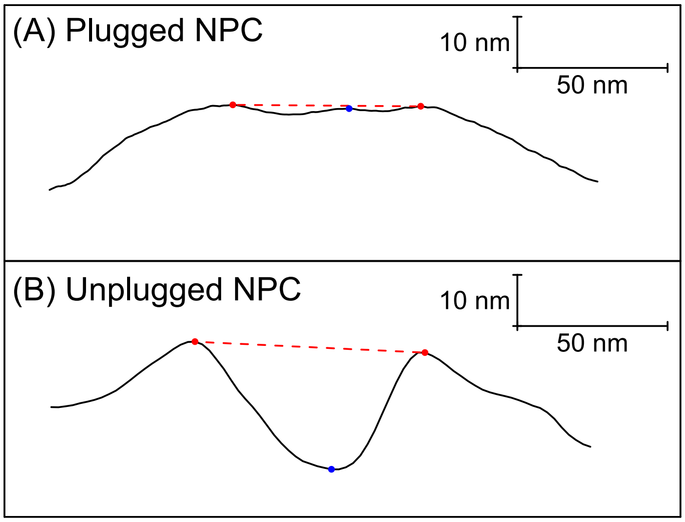

Figure S-2. AFM cross-sections of (A) plugged and (B) unplugged NPCs treated with MIB and NIM, respectively.

Permeability of Fixed and Unfixed NEs. We measured SECM approach curves at MIB- and NIM-treated NEs (Figures S-3A and S-3B, respectively) by using micropipet tips as reported elsewhere[S-2] to demonstrate that the approach curves were nearly identical with (red lines) and without (blue lines) fixation. Experimental approach curves fitted well with the simulated approach curves (circles) to yield the slightly lower permeability of the NIM-treated NE, *k*.[S-3]

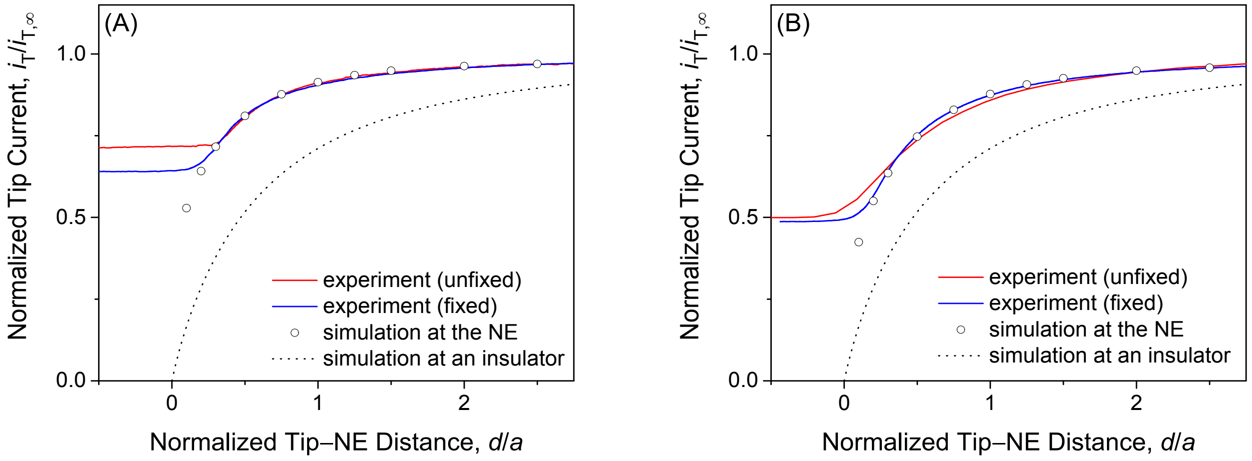

Figure S-3. SECM approach curves of TBA^+ at glass-supported NEs in (A) MIB and (B) NIM with and without glutaraldehyde fixation. Simulated curves at the NE used (a, k) = (0.59 µm, 0.061 cm/s) and (0.45 µm, 0.040 cm/s) for MIB and NIM, respectively, with $RG = 2$.

REFERENCES

(S-1) Chen, R.; Balla, R. J.; Lima, A.; Amemiya, S. Characterization of nanopipet-supported ITIES tips for scanning electrochemical microscopy of single solid-state nanopores. *Anal. Chem.* **2017**, *89*, 9946–9952.

(S-2) Pathirathna, P.; Balla, R. J.; Meng, G.; Wei, Z.; Amemiya, S. Nanoscale electrostatic gating of molecular transport through nuclear pore complexes as probed by scanning electrochemical microscopy. *Chem. Sci.* **2019**, *10*, 7929–7936.

(S-3) Pathirathna, P.; Balla, R. J.; Jantz, D. T.; Kurapati, N.; Gramm, E. R.; Leonard, K. C.; Amemiya, S. Probing high permeability of nuclear pore complexes by scanning electrochemical microscopy: Ca^{2+} effects on transport barriers. *Anal. Chem.* **2019**, *91*, 5446–5454.

(S-4) Eibauer, M.; Pellanda, M.; Turgay, Y.; Dubrovsky, A.; Wild, A.; Medalia, O. Structure and gating of the nuclear pore complex. *Nat. Commun.* **2015**, *6*, 7532.

(S-5) Sakiyama, Y.; Mazur, A.; Kapinos, L. E.; Lim, R. Y. H. Spatiotemporal dynamics of the nuclear pore complex transport barrier resolved by high-speed atomic force microscopy. *Nat. Nanotechnol.* **2016**, *11*, 719–723.
