# Supplementary material for: Nanoscale Quantitative Imaging of Single Nuclear Pore Complexes by Scanning Electrochemical Microscopy
Source: Anal Chem. 2024 Jun 21;96(26):10765–71. doi: 10.1021/acs.analchem.4c01890 (PMC11223102; doi:10.1021/acs.analchem.4c01890)
Supplement: Supplementary file 2 — ac4c01890_si_002.pdf [file ac4c01890_si_002.pdf]

Open NPC

Report date	Apr 10, 2024, 1:54:59 PM

Contents

1. Global Definitions
1.1. Parameters
2. Component 1
2.1. Definitions
2.2. Geometry 1
2.3. Transport of Diluted Species
2.4. Mesh 1
3. Study 1
3.1. Stationary
3.2. Solver Configurations
4. Results
4.1. Data Sets
4.2. Derived Values
4.3. Tables
4.4. Plot Groups

1. Global Definitions

Date	Apr 10, 2024, 1:12:52 PM

Global settings

Name	Single NPC Imaging.mph
Path	E:\Single NPC Imaging.mph
Version	COMSOL Multiphysics 6.2 (Build: 339)
Unit system	SI

Used products

COMSOL Multiphysics

Computer information

CPU	Intel64 Family 6 Model 158 Stepping 13, 8 cores, 15.79 GB RAM
Operating system	Windows 10

1.1. Parameters

Parameters 1

Name	Expression	Value	Description
d	0 [m]	0 m	
l	0 [m]	0 m	
depth	-2.28	−2.28	

2. Component 1

Date	May 23, 2017, 12:42:46 PM

Settings

Description	Value
Unit system	Same as global system (SI)
Geometry shape function	Automatic
Avoid inverted elements by curving interior domain elements	Off

Spatial frame coordinates

First	Second	Third
x	y	z

Material frame coordinates

First	Second	Third

X	Y	Z

Geometry frame coordinates

First	Second	Third
Xg	Yg	Zg

Mesh frame coordinates

First	Second	Third
Xm	Ym	Zm

2.1. Definitions

2.1.1. Probes

Boundary Probe 1

Probe type	Boundary probe

Selection

Geometric entity level	Boundary
Selection	Geometry geom1: Dimension 2: Boundary 12

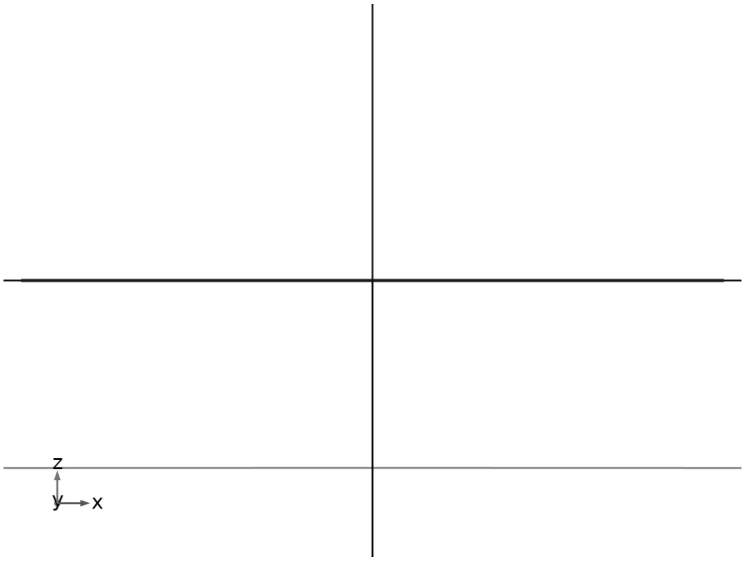

Selection

Expression

Description	Value
Expression	intop1(tds.ndflux_c)
Table and plot unit	mol/s
Description	intop1(tds.ndflux_c)

Table and window settings

Description	Value
Output table	Probe Table 1
Plot window	Probe Plot 1

2.1.2. Nonlocal Couplings

Integration 1

Coupling type	Integration
Operator name	intop1

Selection

Geometric entity level	Boundary

Selection	Geometry geom1: Dimension 2: Boundary 12

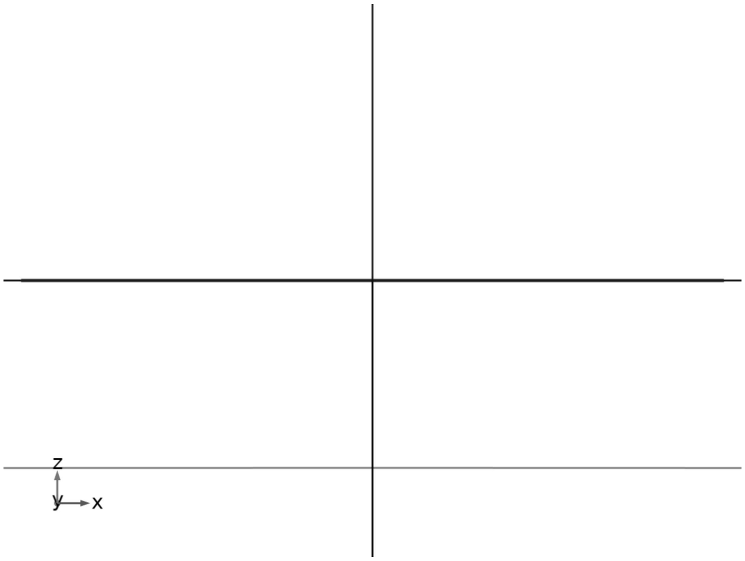

Selection

2.1.3. Coordinate Systems

Boundary System 1

Coordinate system type	Boundary system
Tag	sys1

Coordinate names

First	Second	Third
t1	t2	n

2.2. Geometry 1

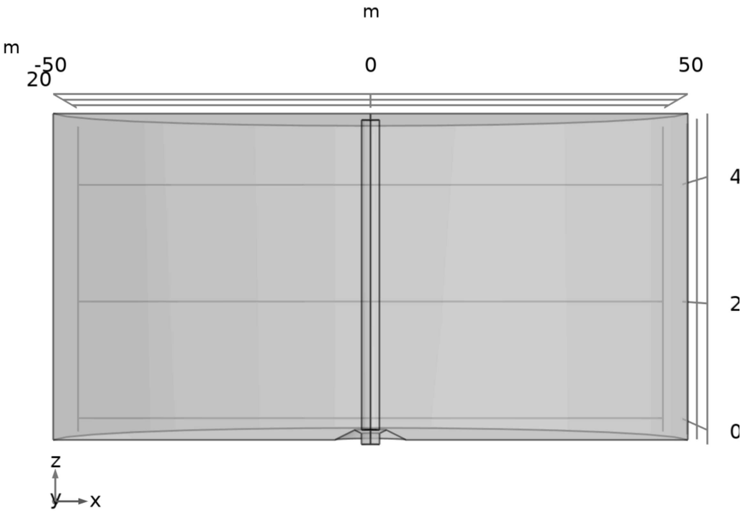

Geometry 1

Units

Length unit	m
Angular unit	deg

Geometry statistics

Description	Value
Space dimension	3

Number of domains	1
Number of boundaries	21
Number of edges	50
Number of vertices	31

2.2.1. Work Plane 1 (wp1)

Plane definition

Description	Value
Plane	xz - plane

Unite objects

Description	Value
Unite objects	On

Information

Description	Value
Last build time	< 1 second
Built with	COMSOL 6.2.0.339 (win64), Apr 10, 2024, 1:54:07 PM

Plane Geometry (sequence2D)

Bézier Polygon 1 (b1)

Polygon segments

Description	Value
Control points	{{-1.43, -1.43, -2.5, -5.625, -5.625, -1.43}, {depth, -0.535, 0, -1.5625, depth, depth}}
Degree	{1, 1, 1, 1, 1}
Weights	{1, 1, 1, 1, 1, 1, 1, 1, 1, 1}
Type	Solid

Information

Description	Value
Last build time	Unknown

2.2.2. Revolve 1 (rev1)

General

Description	Value
Work plane	Work Plane 1 (wp1)

Revolution angles

Description	Value
Angles	{0, 2*pi}
Start angle	180

Revolution axis

Description	Value
Point on the revolution axis	{0, 0}
Direction of revolution axis	{0, 1}
Revolution axis	{{0, 0}, {0, 1}}

Information

Description	Value
Last build time	< 1 second
Built with	COMSOL 6.2.0.339 (win64), Apr 10, 2024, 1:54:07 PM

2.2.3. Work Plane 2 (wp2)

Plane definition

Description	Value
Plane	xz - plane

Unite objects

Description	Value
Unite objects	On

Information

Description	Value
Last build time	< 1 second
Built with	COMSOL 6.2.0.339 (win64), Apr 10, 2024, 1:54:07 PM

Plane Geometry (sequence2D)

Bézier Polygon 1 (b1)

Polygon segments

Description	Value
Control points	{{0, -2.8, -2.8, -50, -50, 0, 0}, {depth, depth, -1.5625, -1.5625, 50, 50, depth}}
Degree	{1, 1, 1, 1, 1, 1}
Weights	{1, 1, 1, 1, 1, 1, 1, 1, 1, 1, 1, 1}
Type	Solid

Information

Description	Value
Last build time	Unknown

2.2.4. Revolve 2 (rev2)

General

Description	Value
Work plane	Work Plane 2 (wp2)

Revolution angles

Description	Value
Angles	{0, 2*pi}
Start angle	180

Revolution axis

Description	Value
Point on the revolution axis	{0, 0}
Direction of revolution axis	{0, 1}
Revolution axis	{{0, 0}, {0, 1}}

Information

Description	Value
Last build time	< 1 second
Built with	COMSOL 6.2.0.339 (win64), Apr 10, 2024, 1:54:07 PM

2.2.5. Difference 1 (dif1)

Information

Description	Value
Last build time	< 1 second
Built with	COMSOL 6.2.0.339 (win64), Apr 10, 2024, 1:54:07 PM

2.2.6. Work Plane 3 (wp3)

Unite objects

Description	Value
Unite objects	On

Information

Description	Value
Last build time	< 1 second

Built with	COMSOL 6.2.0.339 (win64), Apr 10, 2024, 1:54:07 PM

Plane Geometry (sequence2D)

Circle 1 (c1)

Position

Description	Value
Position	{l, 0}

Rotation angle

Description	Value
Rotation	360

Size and shape

Description	Value
Radius	1.4
Sector angle	180

Information

Description	Value
Last build time	Unknown

2.2.7. Work Plane 4 (wp4)

Unite objects

Description	Value
Unite objects	On

Information

Description	Value
Last build time	< 1 second
Built with	COMSOL 6.2.0.339 (win64), Apr 10, 2024, 1:54:07 PM

Plane Geometry (sequence2D)

Circle 1 (c1)

Position

Description	Value
Position	{l, 0}

Rotation angle

Description	Value
Rotation	360

Size and shape

Description	Value
Radius	1
Sector angle	180

Information

Description	Value
Last build time	Unknown

2.2.8. Extrude 1 (ext1)

General

Description	Value
Work plane	Work Plane 3 (wp3)

Distances

Distances (m)
49

Scales

Scales xw	Scales yw
1	1

Displacements

Displacements xw (m)	Displacements yw (m)
0	0

Twist angles

Twist angles (deg)
0

Information

Description	Value
Last build time	< 1 second
Built with	COMSOL 6.2.0.339 (win64), Apr 10, 2024, 1:54:07 PM

2.2.9. Difference 2 (dif2)

Information

Description	Value
Last build time	< 1 second
Built with	COMSOL 6.2.0.339 (win64), Apr 10, 2024, 1:54:07 PM

2.2.10. Form Union (fin)

Information

Description	Value
Details	{Formed union of 1 solid object and 1 surface object., Union has 1 domain, 21 boundaries, 50 edges, and 31 vertices.}
Last build time	< 1 second
Built with	COMSOL 6.2.0.339 (win64), Apr 10, 2024, 1:54:07 PM

2.3. Transport of Diluted Species

Used products

COMSOL Multiphysics

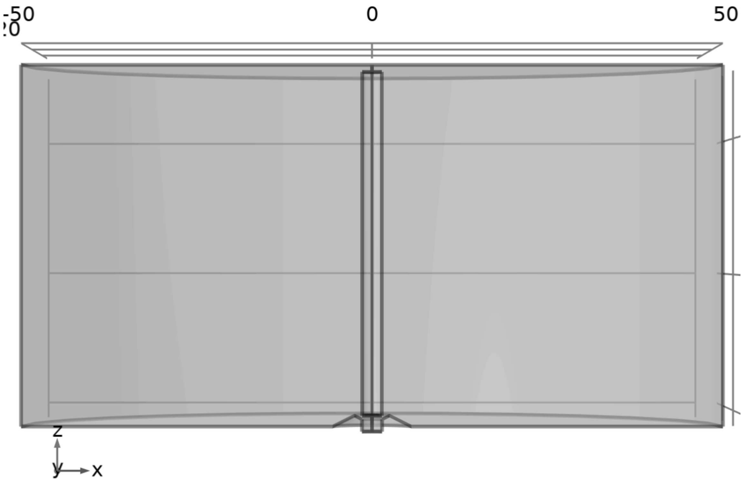

Transport of Diluted Species

Selection

Geometric entity level	Domain
Selection	Geometry geom1: Dimension 3: All domains

Equations

$$\nabla \cdot \mathbf{J}_i + \mathbf{u} \cdot \nabla c_i = R_i$$

$\mathbf{J}_i = -D_i \nabla c_i$

2.3.1. Interface Settings

Discretization

Settings

Description	Value
Concentration	Linear

Settings

Description	Value
Equation form	Study controlled

Species Activity

Settings

Description	Value
Species activity	Ideal

Transport Mechanisms

Settings

Description	Value
Convection	On

2.3.2. Variables

Name	Expression	Unit	Description	Selection	Details
tds.d	1	1	Out-of-plane geometry extension	Global	
tds.f_c	1	1	Activity coefficient	Domain 1	
tds.nx	dnx	1	Normal vector, x-component	Boundaries 1–21	
tds.ny	dny	1	Normal vector, y-component	Boundaries 1–21	
tds.nz	dnz	1	Normal vector, z-component	Boundaries 1–21	
tds.nxmesh	dnxmesh	1	Normal vector (mesh), x-component	Boundaries 1–21	
tds.nymesh	dnymesh	1	Normal vector (mesh), y-component	Boundaries 1–21	
tds.nzmesh	dnzmesh	1	Normal vector (mesh), z-component	Boundaries 1–21	
tds.nxc	nxc/tds.ncLen	1	Normal vector, x-component	Boundaries 1–21	
tds.nyc	nyc/tds.ncLen	1	Normal vector, y-component	Boundaries 1–21	
tds.nzc	nzc/tds.ncLen	1	Normal vector, z-component	Boundaries 1–21	
tds.ncLen	sqrt(nxc^2+nyc^2+nzc^2+eps)	1	Help variable	Boundaries 1–21	
tds.cbf_c	0	mol/(m²·s)	Convective boundary flux	Boundaries 1–21	
tds.u	0	m/s	Velocity field, x-component	Domain 1	
tds.v	0	m/s	Velocity field, y-component	Domain 1	
tds.w	0	m/s	Velocity field, z-component	Domain 1	
tds.R_c	0	mol/(m³·s)	Total rate expression	Domain 1	+ operation
tds.cP_c	0	mol/kg	Concentration species adsorbed to the solid	Domain 1	+ operation
tds.cP_c	0	mol/kg	Concentration species adsorbed to the solid	Boundaries 1–21	+ operation
tds.KP_c	0	m³/kg	Adsorption isotherm, first concentration derivative	Domain 1	+ operation
tds.KP_c	0	m³/kg	Adsorption isotherm, first concentration derivative	Boundaries 1–21	+ operation
tds.Rads_c	0	mol/(m³·s)	Total adsorption rate	Domain 1	+ operation
tds.DiT_c	0	m²/s	Turbulent diffusivity	Domain 1	
tds.cVar_c	c	mol/m³	Species	Boundaries 1–21	
tds.cVar_c	c	mol/m³	Species	Edges 1–50	
tds.cVar_c	c	mol/m³	Species	Points 1–31	
tds.poro	1	1	Porosity	Domain 1	
tds.theta_g	0	1	Gas volume fraction	Domain 1	

tds.theta_l	1	1	Liquid volume fraction	Domain 1	
tds.theta	tds.poro	1	Mobile fluid volume fraction	Domain 1	

2.3.3. Transport Properties 1

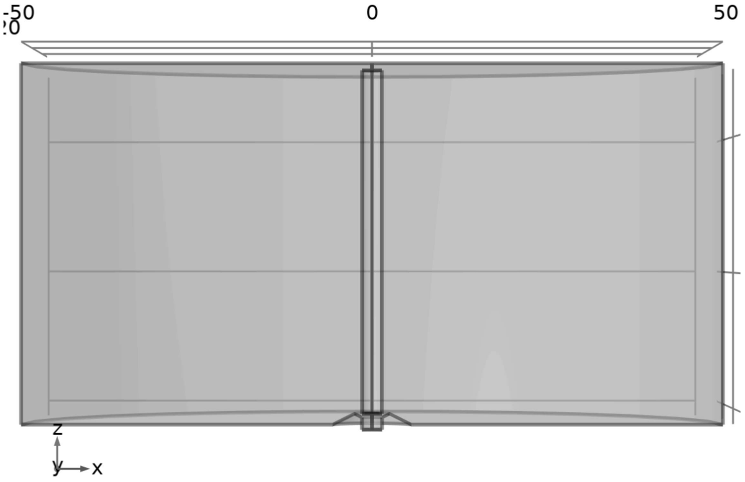

Transport Properties 1

Selection

Geometric entity level	Domain
Selection	Geometry geom1: Dimension 3: All domains

Equations

$$\nabla \cdot \mathbf{J}_i + \mathbf{u} \cdot \nabla c_i = R_i$$

$$\mathbf{J}_i = -D_i \nabla c_i$$

Convection

Settings

Description	Value	Unit
Velocity field	User defined	
Velocity field	{0, 0, 0}	m/s

Diffusion

Settings

Description	Value	Unit
Source	Material	
Material	None	
Diffusion coefficient	User defined	
Diffusion coefficient	1	m²/s

Coordinate System Selection

Settings

Description	Value
Coordinate system	Global coordinate system

Model Input

Settings

Description	Value	Unit

Temperature	User defined	
Temperature	293.15	K

Variables

Name	Expression	Unit	Description	Selection	Details
domflux.cx	tds.dflux_cx*tds.d	mol/(m²·s)	Domain flux, x-component	Domain 1	
domflux.cy	tds.dflux_cy*tds.d	mol/(m²·s)	Domain flux, y-component	Domain 1	
domflux.cz	tds.dflux_cz*tds.d	mol/(m²·s)	Domain flux, z-component	Domain 1	
tds.ndflux_c	tds.bndFlux_c	mol/(m²·s)	Normal diffusive flux	Boundaries 1–21	
tds.ncflux_c	tds.cflux_cx*tds.nxc+tds.cflux_cy*tds.nyc+tds.cflux_cz*tds.nzc	mol/(m²·s)	Normal convective flux	Boundaries 1–21	
tds.ntflux_c	tds.bndFlux_c+tds.cflux_cx*tds.nxc+tds.cflux_cy*tds.nyc+tds.cflux_cz*tds.nzc	mol/(m²·s)	Normal total flux	Boundaries 1–21	
tds.u	model.input.u1	m/s	Velocity field, x-component	Domain 1	Meta
tds.v	model.input.u2	m/s	Velocity field, y-component	Domain 1	Meta
tds.w	model.input.u3	m/s	Velocity field, z-component	Domain 1	Meta
tds.bndFlux_c	-dflux_spatial(c)/tds.d	mol/(m²·s)	Boundary flux	Boundaries 1–21	Meta
tds.DF_cxx	1[m^2/s]	m²/s	Fluid diffusion coefficient, xx-component	Domain 1	
tds.DF_cyx	0	m²/s	Fluid diffusion coefficient, yx-component	Domain 1	
tds.DF_czx	0	m²/s	Fluid diffusion coefficient, zx-component	Domain 1	
tds.DF_cxy	0	m²/s	Fluid diffusion coefficient, xy-component	Domain 1	
tds.DF_cyy	1[m^2/s]	m²/s	Fluid diffusion coefficient, yy-component	Domain 1	
tds.DF_czy	0	m²/s	Fluid diffusion coefficient, zy-component	Domain 1	
tds.DF_cxz	0	m²/s	Fluid diffusion coefficient, xz-component	Domain 1	

tds.DF_cyz	0	m²/s	Fluid diffusion coefficient, yz-component	Domain 1	
tds.DF_czz	1[m^2/s]	m²/s	Fluid diffusion coefficient, zz-component	Domain 1	
tds.D_cxx	tds.DF_cxx+tds.DiT_c	m²/s	Diffusion coefficient, xx-component	Domain 1	+ operation
tds.D_cyx	tds.DF_cyx	m²/s	Diffusion coefficient, yx-component	Domain 1	+ operation
tds.D_czx	tds.DF_czx	m²/s	Diffusion coefficient, zx-component	Domain 1	+ operation
tds.D_cxy	tds.DF_cxy	m²/s	Diffusion coefficient, xy-component	Domain 1	+ operation
tds.D_cyy	tds.DF_cyy+tds.DiT_c	m²/s	Diffusion coefficient, yy-component	Domain 1	+ operation
tds.D_czy	tds.DF_czy	m²/s	Diffusion coefficient, zy-component	Domain 1	+ operation
tds.D_cxz	tds.DF_cxz	m²/s	Diffusion coefficient, xz-component	Domain 1	+ operation
tds.D_cyz	tds.DF_cyz	m²/s	Diffusion coefficient, yz-component	Domain 1	+ operation
tds.D_czz	tds.DF_czz+tds.DiT_c	m²/s	Diffusion coefficient, zz-component	Domain 1	+ operation
tds.Dav_c	(tds.D_cxx+tds.D_cyy+tds.D_czz)/3	m²/s	Average diffusion coefficient	Domain 1	
tds.tflux_cx	tds.dflux_cx+tds.cflux_cx	mol/(m²·s)	Total flux, x-component	Domain 1	+ operation
tds.tflux_cy	tds.dflux_cy+tds.cflux_cy	mol/(m²·s)	Total flux, y-component	Domain 1	+ operation
tds.tflux_cz	tds.dflux_cz+tds.cflux_cz	mol/(m²·s)	Total flux, z-component	Domain 1	+ operation
tds.dfluxMag_c	sqrt(tds.dflux_cx^2+tds.dflux_cy^2+tds.dflux_cz^2)	mol/(m²·s)	Diffusive flux magnitude	Domain 1	
tds.tfluxMag_c	sqrt(tds.tflux_cx^2+tds.tflux_cy^2+tds.tflux_cz^2)	mol/(m²·s)	Total flux magnitude	Domain 1	
tds.dpflux_cx	0	mol/(m²·s)	Dispersive flux, x-component	Domain 1	
tds.dpflux_cy	0	mol/(m²·s)	Dispersive flux, y-component	Domain 1	

tds.dpflux_cz	0	mol/(m²·s)	Dispersive flux, z-component	Domain 1	
tds.c_material	c*spatial.detF	mol/m³	Concentration	Domain 1	
tds.dflux_cx	-tds.D_cxx*cx-tds.D_cxy*cy-tds.D_cxz*cz	mol/(m²·s)	Diffusive flux, x-component	Domain 1	+ operation
tds.dflux_cy	-tds.D_cyx*cx-tds.D_cyy*cy-tds.D_cyz*cz	mol/(m²·s)	Diffusive flux, y-component	Domain 1	+ operation
tds.dflux_cz	-tds.D_czx*cx-tds.D_czy*cy-tds.D_czz*cz	mol/(m²·s)	Diffusive flux, z-component	Domain 1	+ operation
tds.grad_cx	cx	mol/m⁴	Concentration gradient, x-component	Domain 1	
tds.grad_cy	cy	mol/m⁴	Concentration gradient, y-component	Domain 1	
tds.grad_cz	cz	mol/m⁴	Concentration gradient, z-component	Domain 1	
tds.cflux_cx	c*tds.u	mol/(m²·s)	Convective flux, x-component	Domain 1	
tds.cflux_cy	c*tds.v	mol/(m²·s)	Convective flux, y-component	Domain 1	
tds.cflux_cz	c*tds.w	mol/(m²·s)	Convective flux, z-component	Domain 1	
tds.cfluxMag_c	sqrt(tds.cflux_cx^2+tds.cflux_cy^2+tds.cflux_cz^2)	mol/(m²·s)	Convective flux magnitude	Domain 1	
tds.Rlin_c	0	1/s	Linear source term coefficient	Domain 1	+ operation
tds.Res_c	tds.u*cx+tds.v*cy+tds.w*cz-c*tds.Rlin_c-tds.R_c	mol/(m³·s)	Equation residual	Domain 1	

Shape functions

Name	Shape function	Unit	Description	Shape frame	Selection
c	Lagrange (Linear)	mol/m³	Concentration	Material	Domain 1

Weak Expressions

Weak expression	Integration order	Integration frame	Selection
(tds.dflux_cx*test(cx)+tds.dflux_cy*test(cy)+tds.dflux_cz*test(cz))*tds.d	2	Material	Domain 1
-(tds.u*cx+tds.v*cy+tds.w*cz)*test(c)*(isScalingSystemDomain==0)*tds.d	2	Material	Domain 1
tds.cbf_c*test(c)*tds.d	2	Material	Boundaries 1–21
tds.streamline*(isScalingSystemDomain==0)*tds.d	2	Material	Domain 1
tds.crosswind*(isScalingSystemDomain==0)*tds.d	4	Material	Domain 1

2.3.4. No Flux 1

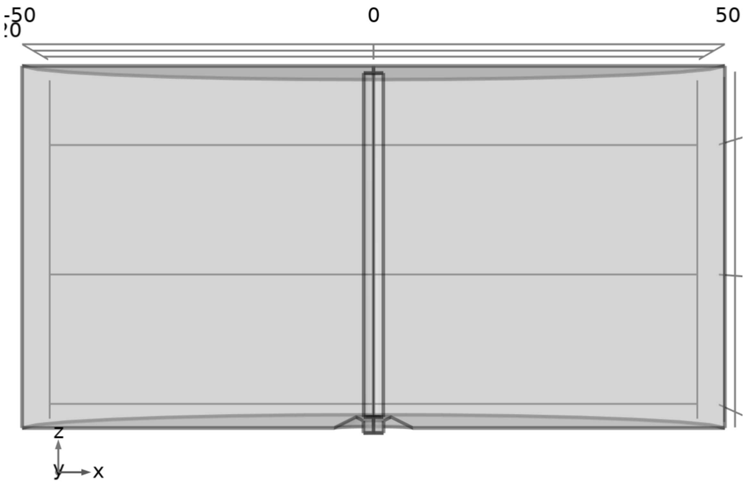

No Flux 1

Selection

Geometric entity level	Boundary
Selection	Geometry geom1: Dimension 2: All boundaries

Equations

$$-\mathbf{n} \cdot \mathbf{J}_i = 0$$

Convection

Settings

Description	Value
Include	Off

2.3.5. Initial Values 1

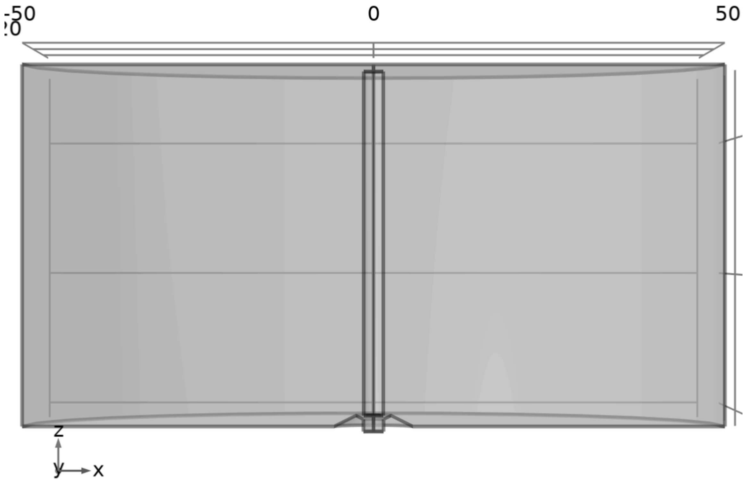

Initial Values 1

Selection

Geometric entity level	Domain
Selection	Geometry geom1: Dimension 3: All domains

Initial Values

Settings

Description	Value	Unit
Concentration	1	mol/m³

Variables

Name	Expression	Unit	Description	Selection	Details
tds.c0_c	1	mol/m³	Concentration	Domain 1	+ operation

2.3.6. Concentration 1

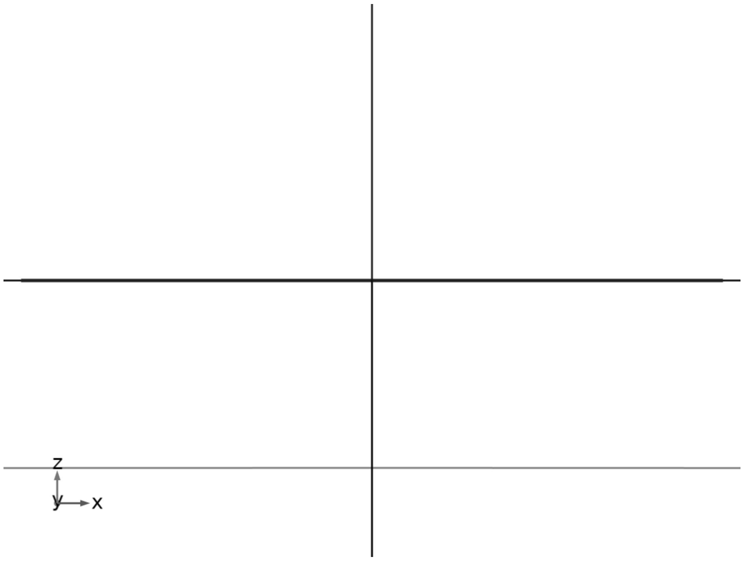

Concentration 1

Selection

Geometric entity level	Boundary
Selection	Geometry geom1: Dimension 2: Boundary 12

Equations

$$c_i = c_{0,j}$$

Concentration

Settings

Description	Value	Unit
Species c	On	
Concentration	0	mol/m³

Variables

Name	Expression	Unit	Description	Selection	Details
tds.c0_c	0	mol/m³	Concentration	Boundary 12	+ operation
tds.conc1.nmflow_c	tds.conc1.int(tds.ntflux_c)*tds.d	mol/s	Normal molar flow rate	Global	

Constraints

Constraint	Constraint force	Shape function	Selection	Details
-tds.cVar_c+tds.c0_c	test(-tds.cVar_c+tds.c0_c)	Lagrange (Linear)	Boundary 12	Elemental

2.3.7. Concentration 2

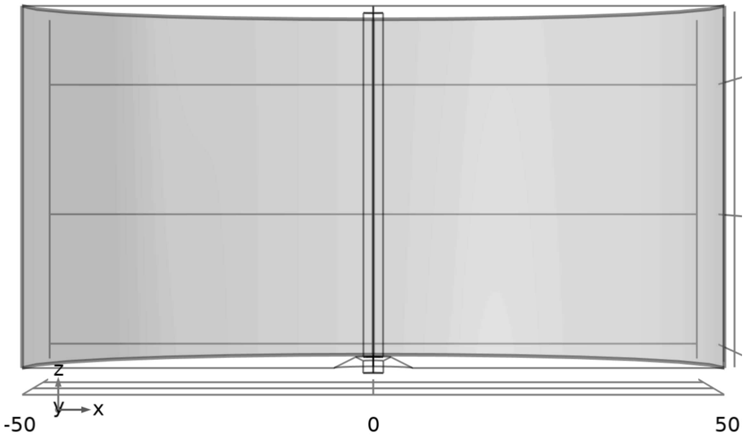

Concentration 2

Selection

Geometric entity level	Boundary
Selection	Geometry geom1: Dimension 2: Boundaries 2, 21

Equations

$$c_i = c_{0,i}$$

Concentration

Settings

Description	Value	Unit
Species c	On	
Concentration	1	mol/m³

Variables

Name	Expression	Unit	Description	Selection	Details
tds.c0_c	1	mol/m³	Concentration	Boundaries 2, 21	+ operation
tds.conc2.nmflow_c	tds.conc2.int(tds.ntflux_c)*tds.d	mol/s	Normal molar flow rate	Global	

Constraints

Constraint	Constraint force	Shape function	Selection	Details
-tds.cVar_c+tds.c0_c	test(-tds.cVar_c+tds.c0_c)	Lagrange (Linear)	Boundaries 2, 21	Elemental

2.4. Mesh 1

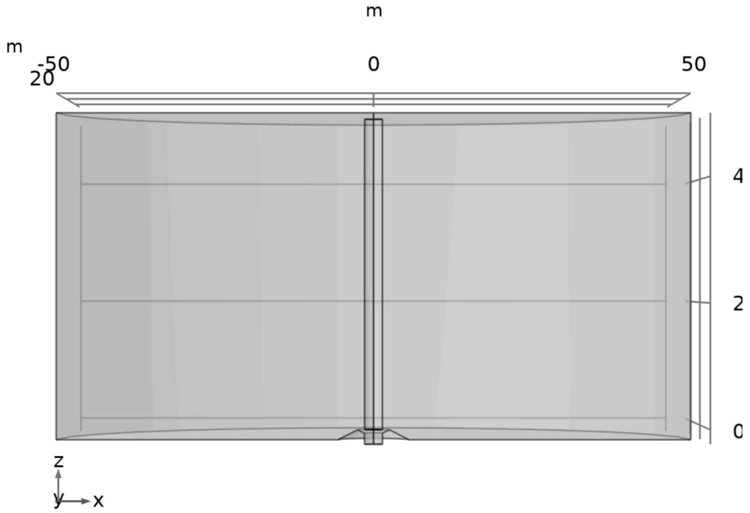

Mesh 1

Mesh statistics

Description	Value
Status	Empty mesh
Mesh vertices	0

2.4.1. Size (size)

Settings

Description	Value
Maximum element size	2
Minimum element size	0.02
Curvature factor	0.2
Maximum element growth rate	1.3
Predefined size	Extremely fine

2.4.2. Free Tetrahedral 1 (ftet1)

Selection

Geometric entity level	Domain
Selection	Remaining

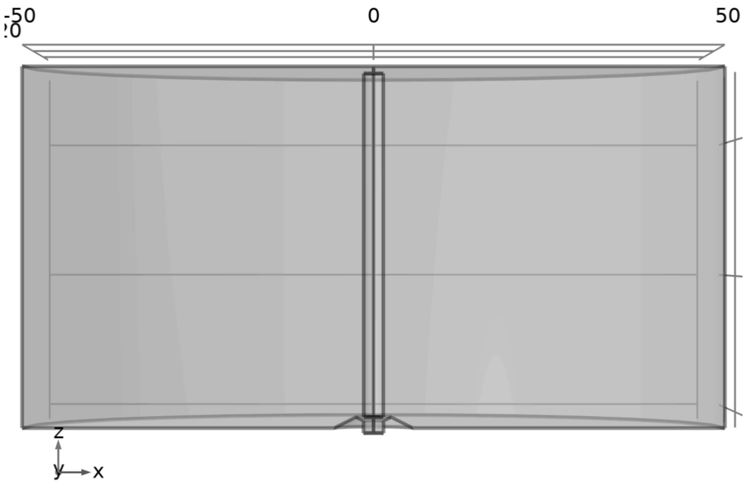

Free Tetrahedral 1

Information

Description	Value
Method	Delaunay (legacy version)
Last build time	7 seconds
Built with	COMSOL 6.1.0.357 (win64), Apr 10, 2024, 1:16:14 PM

Distribution 1 (dis1)

Selection

Geometric entity level	Edge
Selection	Geometry geom1: Dimension 1: Edges 19–20, 24, 29

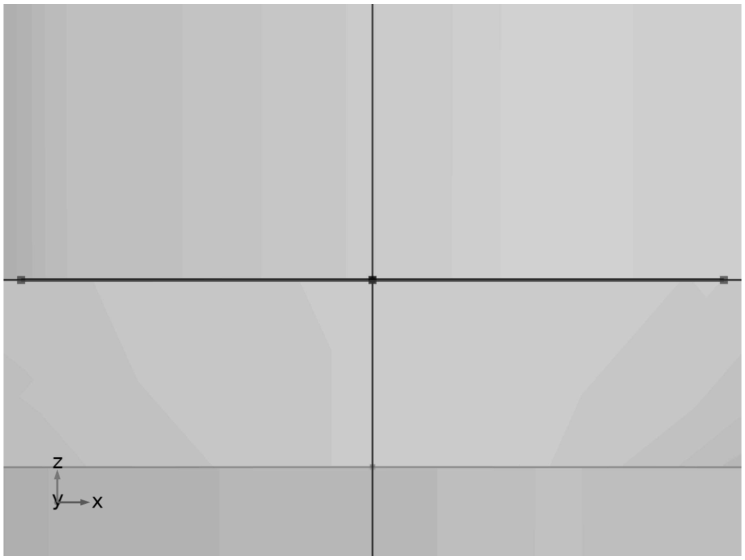

Distribution 1

Settings

Description	Value
Number of elements	100

3. Study 1

Computation information

Computation time	3 s

3.1. Stationary

Study settings

Description	Value
Include geometric nonlinearity	Off

Physics and variables selection

Physics interface	Solve for	Equation form
Transport of Diluted Species (tds)	On	Automatic (Stationary)

Store in output

Interface	Output	Selection
Transport of Diluted Species (tds)	Physics controlled	

Mesh selection

Component	Mesh
Component 1	Mesh 1

3.2. Solver Configurations

3.2.1. Solution 1

Compile Equations: Stationary (st1)

Study and step

Description	Value
Use study	Study 1
Use study step	Stationary

Log

<---- Compile Equations: Stationary in Study 1/Solution 1 (sol1) ---------------
Started at Apr 10, 2024, 1:16:14 PM.
Geometry shape function: Linear Lagrange
Running on Intel64 Family 6 Model 158 Stepping 13, GenuineIntel.
Using 1 socket with 8 cores in total on DESKTOP-8IKUPMM.
Available memory: 16.17 GB.
Time: 1 s.
Physical memory: 1.57 GB
Virtual memory: 1.55 GB
Ended at Apr 10, 2024, 1:16:16 PM.
----- Compile Equations: Stationary in Study 1/Solution 1 (sol1) -------------->

Dependent Variables 1 (v1)

General

Description	Value
Defined by study step	Step 1: Stationary

Log

<---- Dependent Variables 1 in Study 1/Solution 1 (sol1) -----------------------
Started at Apr 10, 2024, 1:16:16 PM.
Solution time: 0 s.
Physical memory: 1.52 GB
Virtual memory: 1.48 GB
Ended at Apr 10, 2024, 1:16:16 PM.
----- Dependent Variables 1 in Study 1/Solution 1 (sol1) ---------------------->

Concentration (comp1.c) (comp1_c)

General

Description	Value
Field components	comp1.c
Internal variables	{comp1.uflux.c, comp1.dflux.c}

Stationary Solver 1 (s1)

General

Description	Value
Defined by study step	Step 1: Stationary

Log

<---- Stationary Solver 1 in Study 1/Solution 1 (sol1) ------------------------
Started at Apr 10, 2024, 1:16:16 PM.
Linear solver
Number of degrees of freedom solved for: 107817 (plus 16994 internal DOFs).
Symmetric matrices found.
Format not changed since SOR line uses nonsymmetric storage.
Scales for dependent variables:
Concentration (comp1.c): 1
Orthonormal null-space function used.

```
Iter    SolEst    Damping    Stepsize #Res #Jac #Sol LinIt   LinErr   LinRes
   1       3.1  1.0000000         3.1    1    1    1    10  0.00039  5.5e-06
```

Solution time: 2 s.
Physical memory: 1.61 GB
Virtual memory: 1.58 GB
Ended at Apr 10, 2024, 1:16:17 PM.
----- Stationary Solver 1 in Study 1/Solution 1 (sol1) ---------------------->

Advanced (aDef)

Assembly settings

Description	Value
Reuse sparsity pattern	On

Fully Coupled 1 (fc1)

General

Description	Value
Linear solver	AMG, concentrations (tds)

Method and termination

Description	Value
Initial damping factor	0.01
Minimum damping factor	1E-6
Maximum number of iterations	50

AMG, concentrations (tds) (i1)

General

Description	Value
Nonlinear-based error norm	On
Maximum number of iterations	1000

Multigrid 1 (mg1)

General

Description	Value
Solver	Smoothed aggregation AMG
Maximum number of DOFs at coarsest level	50000
Construct prolongators componentwise	On
Prolongator smoothing	Off

Presmoother (pr)

SOR Line 1 (sl1)

Main

Description	Value
Sweep type	SSOR
Number of iterations	1
Relaxation factor	0.7

Secondary

Description	Value
Relaxation factor	0.5

Postsmoother (po)

SOR Line 1 (sl1)

Main

Description	Value
Sweep type	SSOR
Number of iterations	1
Relaxation factor	0.7

Secondary

Description	Value

Relaxation factor	0.5

Coarse Solver (cs)

Direct 1 (d1)

General

Description	Value
Solver	PARDISO
Pivoting perturbation	1E-13

4. Results

4.1. Data Sets

4.1.1. Study 1/Solution 1

Solution

Description	Value
Solution	Solution 1 (sol1)
Component	Component 1 (comp1)

4.1.2. Probe Solution 2

Solution

Description	Value
Solution	Solution 1 (sol1)
Component	Component 1 (comp1)

4.1.3. Boundary Probe 1

Selection

Geometric entity level	Boundary
Selection	Geometry geom1: Dimension 2: Boundary 12

Data

Description	Value
Dataset	Probe Solution 2 (sol1)

Settings

Description	Value
Method	Integration
Integration order	4
Integration order	On

4.2. Derived Values

4.2.1. Boundary Probe 1

Output

Evaluated in	Probe Table 1

Data

Description	Value
Dataset	Boundary Probe 1

Expressions

Expression	Unit	Description
intop1(tds.ndflux_c)	mol/s	intop1(tds.ndflux_c)

4.3. Tables

4.3.1. Probe Table 1

intop1(tds.ndflux_c) (mol/s), Boundary Probe 1
1.4427

4.4. Plot Groups

4.4.1. Concentration (tds)

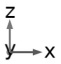

Slice: Concentration (mol/m3)

4.4.2. Concentration (tds) 1

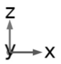

Surface: Concentration (mol/m3)

4.4.3. Probe Plot Group 3

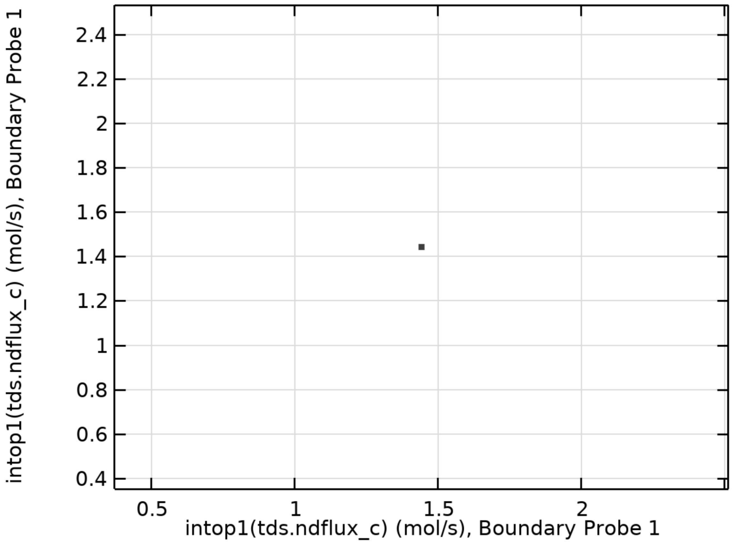
intop1(tds.ndflux_c) (mol/s), Boundary Probe 1
2.4
2.2
2
1.8
1.6
1.4
1.2
1
0.8
0.6
0.4
0.5
1
1.5
2
intop1(tds.ndflux_c) (mol/s), Boundary Probe 1
